# Improving the Accuracy of the Diffusion Model in Highly Absorbing Media

**DOI:** 10.1155/2007/38168

**Published:** 2007-08-30

**Authors:** Alexander X. Cong, Haiou Shen, Wenxiang Cong, Ge Wang

**Affiliations:** Biomedical Imaging Division, School of Biomedical Engineering and Sciences, Virginia Polytechnic Institute and State University, 1880 Pratt Drive, Blacksburg, VA 24061, USA

## Abstract

The diffusion approximation of the Boltzmann transport equation is most commonly used for describing the photon propagation in turbid media. It produces satisfactory results in weakly absorbing and highly scattering media, but the accuracy lessens with the decreasing albedo. In this paper, we presented a method to improve the accuracy of the diffusion model in strongly absorbing media by adjusting the optical parameters. Genetic algorithm-based optimization tool is used to find the optimal optical parameters. The diffusion model behaves more closely to the physical model with the actual optical parameters substituted by the optimized optical parameters. The effectiveness of the proposed technique was demonstrated by the numerical experiments using the Monte Carlo simulation data as measurements.

## 1. INTRODUCTION

The optical tomography techniques such as bioluminescence tomography (BLT), fluorescent tomography (FMT), and diffusion optical tomography (DOT) have attracted increasing research attentions in recent years. One of the common core issues of optical imaging modalities is how to model the light propergation in biological tissues properly. Monte Carlo simulation, which has numerous successful applications in other fields, was introduced to study the light interaction with tissue [1, 2]. Although it is a rigorous model for forward problem of photon transport [2], due to its stochastic nature, the excessive computational requirement makes it an improper choice for inverse problems. On the other hand, the Boltzmann transport equation is able to model the photon propergation deterministicly and accurately in tissue [3], but it too has a high computational complexity. Several methods were proposed to solve the transport equation, such as discrete ordinates [4, 5] and spherical harmonics expansion [6], but applying transport equation in 3D remains challenging in pratice. To reduce the complexity, diffusion approximation (DA) was introduced and widely applied as a photon propergation model in various optical tomography modalities [7–9]. DA is computationally efficient and almost as accurate as the transport equation in weakly absorbing media. Unfortunately, for albedos $\mu_{s}^{\prime }/\mu_{a}<10,$ DA is no longer able to describe the photon propagation accurately [10, 11]. The relatively strong photon absorptions are often resulted from the shorter wavelength of the broad emission spectrum of a reporter gene, such as the luciferase used in BLT, which has emission peaks between 538 to 570 nm [12]. It was reported that performing BLT at a shorter wavelength helps to reduce the ill posedness of the reconstruction [11]. Therefore, it is important that photon propagation in strongly absorbing media could be modeled.

In this paper, we proposed an optical parameter adjustment technique to alleviate the inaccuracy of DA in highly absorbing media. In our approach, we make the diffusion model adjustable in the sense that the optical parameters, which are usually considered as the known properties, are interpreted as variables. The accuracy of the model is no longer solely controlled by the formulation of DA, but also by the adjustable optical parameters. The optical parameters that minimize the error between the solution of DA and the simulated MC data make the model more accurate than use the intrinsic optical properties directly. This technique is discussed in Section 2.

## 2. SIMULATION METHODS

In this section, we give a brief overview of the two simulation methods used in the numerical studies: the Monte Carlo simulation and the finite element solution of the diffusion equation, and discuss the optical parameters adjustment technique.

### 2.1. Monte Carlo simulation

**Figure 1:**
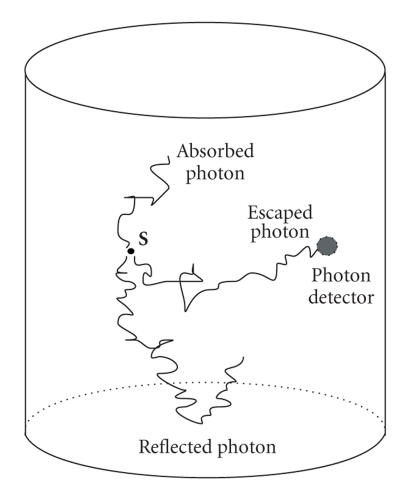
The Monte Carlo process of photon propagation in tissue.

Monte Carlo (MC) simulation models each individual photon's physical interactions with the medium as a stochastic process. As a large number of such stochastic processes of photon propagation are simulated, the signal detected is statistically meaningful and very close to the physical experiment counterpart. MC result can be legitimately considered as the low-noise version of the actual physical measurement. Therefore, we use MC method to produce measurements in numerical experiments. The Monte Carlo process consists of three parts: the photon absorption, the photon scattering and the internal reflection at the boundaries, as illustrated in Figure 1. The absorption of photon for each step can be expressed by [2,3,13]

(1) $$\Delta W=\frac{\mu_{a}}{\mu_{a}+\mu_{s}}W,$$

where W is the weight of the photon packet. The scattering of the photon is governed by Henyey-Greenstein (HG) phase function, which is considered as the most appropriate phase function for the photon propergation in tissue. The HG phase function is given by [2, 3]

(2) $$p\left(\cos\;\theta \right)=\frac{1-g^{2}}{2{\left(1+g^{2}-2g\cos\theta \right)}^{3/2}},$$

where θ is the deflection angle, and g the anisotropy. The internal reflectance rate due to the refrective index mismatch at the tissue boundary for unpolarized incident light is given by the Fresnel's formulas [2, 3]:

(3) $$R\left(\vartheta_{i}\right)=\frac{1}{2}\left[\frac{\sin^{2}\left(\vartheta_{i}-\vartheta_{t}\right)}{\sin^{2}\left(\vartheta_{i}+\vartheta_{t}\right)}+\frac{\tan^{2}\left(\vartheta_{i}-\vartheta_{t}\right)}{\tan^{2}\left(\vartheta_{i}+\vartheta_{t}\right)}\right],$$

where $\vartheta_{i}$ and $\vartheta_{t}$ are the incident and transmit angles, respectively. The incident and transmit angles obey the Snell's law

(4) $$\frac{\sin\vartheta_{i}}{\sin\vartheta_{t}}=\frac{n_{t}}{n_{i}},$$

where $n_{i}$ and $n_{t}$ are the refractive indices for both sides of the boundary, respectively.

We programmed MCsim [14], a Monte Carlo simulator, for the photon propagation for the numerical experiment. Our MC simulator can handle several types of 3D geometrical phantoms such as cylinders and ellipsoids. By combining cylinders and ellipsoids, which can represent different mouse organs, the heterogeneous phantom mimicking the real mouse is created. The MC simulation based on such heterogeneous numerical phantom is similar to the in vivo mouse experiment. The efficiency of the MC simulation is enhanced through the parallel computing and fast random array generateration.

### 2.2. Diffusion model

The transport equation accurately characterizes the photon propergation in biological tissue. Due to its complexity in 3D, the diffusion approximation of the transport equation is often used instead in tissue with high albedo. The diffusion equation in steady state is given by [3, 8]

(5) $$-\nabla \cdot \left(D\left(r\right)\nabla \varphi \left(r\right)\right)+\mu_{a}\varphi \left(r\right)=S\left(r\right),\;\;\;\;r\in \Omega$$

and the Robin boundary condition is applied [8, 15]:

(6) φ(r)+2Θ(r)D(r)n⋅∇φ(r)=0,    r∈∂Ω,

where r is the position vector, φ the photon fluence, S the source power density, Ω the internal region, n the normal to the boundary ∂Ω, and Θ the boundary mismatch factor, which is given by (1+R)/(1−R), and R can be approximated by $R\approx -1.4399n_{i}^{-2}+0.7099n_{i}^{-1}+0.6681+0.0636n_{i}$ [16]. $\mu_{a}$ and D are the absorption and diffusion coefficient, respectively. D can be decomposed to the expression of $\mu_{a}$ and the reduced scattering coefficient $\mu_{s}^{\prime }$:

(7) $$D=\frac{1}{3\left(\mu_{a}+\mu_{s}^{\prime }\right)}.$$

To solve the diffusion equation, we apply the finite element method and transform the problem into a system of discrete linear equations [8]

(8) Aφ=S,

where A is the weight matrix and S the source power distribution vector. We take the boundary fluence φ(r), r∈∂Ω as measurement to compare with the MC simulated measurement.

### 2.3. Optical parameters adjustment

The simulation of photon progation in tissue not only requires an appropriate model, the optical parameters $\mu_{a}$ and $\mu_{s}^{\prime }$ also play an important role. As we mentioned before, the diffusion model that uses the tissue's intrinsic optical properties as optical parameters is not suitable to describe the photon propagation in strongly absorbing media. However, in pratice we are often only interested in how the photons propagate in tissue without noticing the optical parameters used. For example, the BLT and FMT focus on the reconstruction of the light source distribution. Thus, the optical parameters can be taken as variables to improve the accuracy of the DA model. In this perspective, the accuracy improvement task becomes a typical parameter estimation problem: finding the best parameters that minimize the error of DA at the surface. Therefore, by optimizing the optical parameters, the solution of DA may fit the real measurement better and consequently improve the accuracy of the model.

Before we can find the optimal optical parameters, we first define the error metric, which is simply the average of the relative errors

(9) $$ɛ\left(\mu_{a},\mu_{s}^{\prime }\right)=\frac{\sum_{d}\left|\left(\varphi -\varphi^{\prime }\right)/\varphi \right|}{n_{d}},$$

where ɛ represents the error, φ the MC simulated power density (i.e., fluence) at a detector, $\varphi^{\prime }$ the diffusion model finite element solution of surface power densities at each detector, d the detectors, and $n_{d}$ the number of detectors. This simple error metric evenly weights the strong and weak signals, therefore, the optimization result does not depend on the location of the light source.

It is clear that the error ɛ can be expressed as a function of the optical parameters $\mu_{a}$ and $\mu_{s}^{\prime }$. The optimal optical parameters are found by minimizing the error, as in the following equation:

(10) $$\left\{\mu_{a}^{\text{adj}},\mu_{s}^{\prime \text{adj}}\right\}=\underset{\mu_{a},\;\mu_{s}^{\prime }}{\text{arg}\;\text{min}}\left|ɛ\left(\mu_{a},\mu_{s}^{\prime }\right)\right|.$$

To solve this parameter estimation in (10), we use a genetic algorithm (GA). The major advantages of GA over the deterministic gradient methods are the initial values have very little impact on the optimization result, and the solution will not trapped in a local optima [17]. Although there is no way to know if GA reaches the exact global optima, this stochastic optimization method always produces a sufficiently good solution if a large number of generations (i.e., iterations) and a proper population size are applied. The optimized optical parameters $\mu_{a}^{\text{adj}}$ and $\mu_{s}^{\prime \text{adj}}$ help to improve the accuracy of the diffusion equation (5), as shown in Section 3.

## 3. NUMERICAL EXPERIMENTS AND RESULTS

### 3.1. Optical parameter optimization

We performed the optical parameter optimization on a sphere with 7 mm radius. In Cartesian coordinate, the center of the sphere was the origin. In order to obtain the finite element-based solution of diffusion approximation (DA), the sphere was discretized into 5539 nodes and 28 607 tetrahedra. For the convenience of comparing MC simulation data with the finite element solution of DA, all 1452 surface nodes were used as detectors. For the Monte Carlo simulation, each detector integrated the escaping photons within 0.7 mm radius, while the solution of DA was produced directly at each surface node. The measurement was normalized and served as a description of the boundary power distribution rather than the actual power density.

We placed a single isotropic point source 1 mm away from the origin, and set the source power to 0.313 picowatt, which is equal to the energy of a million photons per second under 635 nm wavelength. At this source location, we performed the optical parameter adjustment for two media: medium one had $\mu_{a}=0.2$ mm^−1^ and medium two had $\mu_{a}=0.35$ mm^−1^, the $\mu_{s}^{\prime }=1.05$ mm^−1^ and the tissue refractive indices are 1.37 for both materials. The albedo of these two media were 5.25 and 3, respectively. We expected that the solutions of DA would have noticeable inaccuracy in these media. We used the genetic algorithm toolbox (*gatool*) in MATLAB to solve (10). The important parameters used in GA are listed in Table 1.

The resultant optimal optical parameters are listed in Table 2.

### 3.2. Accuracy improvement results

**Figure 2:**
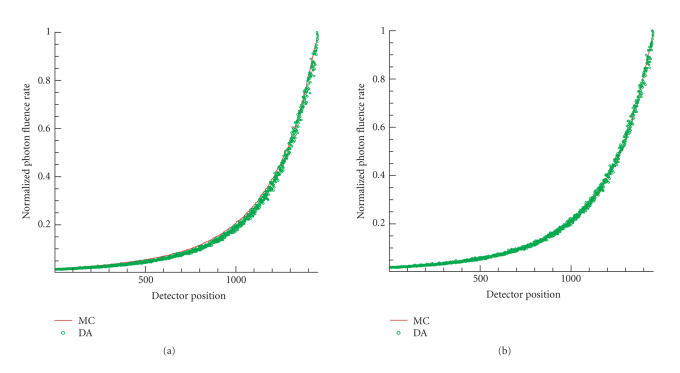
The comparison between solutions of DA using the the unadjusted and the optimal optical parameters in a medium with $\mu_{a}=0.2$ mm^−1^ and $\mu_{s}^{\prime }=1.05$ mm^−1^. The light source was 1 mm from the origin. (a) Solution of DA with unadjusted optical parameters versus MC data. (b) Solution of DA with optimized optical parameters versus MC data. The detector positions were sorted in the increasing order of the fluence rate of MC data.

**Figure 3:**
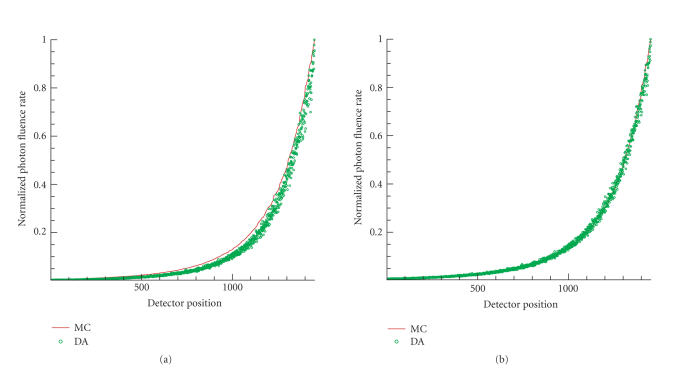
The comparison between solutions of DA using the the unadjusted and the optimal optical parameters in a medium with $\mu_{a}=0.35$ mm^−1^ and $\mu_{s}^{\prime }=1.05$ mm^−1^. The light source was 1 mm from the origin. (a) Solution of DA with unadjusted optical parameters versus MC data. (b) Solution of DA with optimized optical parameters versus MC data. The detector positions were sorted in the increasing order of the fluence rate of MC data.

Using the optimized parameters in Table 2, the results of the accuracy improvements of DA are plotted in Figures 2 and 3, with respect to the two media.

We tested the effectiveness of the optimized optical parameters by solving the DA with different light source locations in the sphere. The accuracy enhancement results regarding different light source locations are listed in Table 3. The error metric in Table 3 was according to (9). The results show that the accuracy improvement effect was stable for different light source locations, and has little dependency on the location of the light source where the optical parameter optimization was performed.

**Figure 4:**
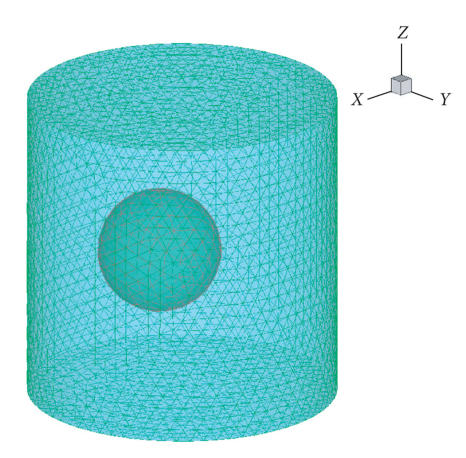
The finite element model of the heterogeneous phantom.

To further test the effectiveness of the proposed method, we constructed a heterogeneous phantom, as in Figure 4. The outer cylinder had a height of 20 mm and a radius of 10 mm. The geometrical center of the cylinder was at the origin. The inner sphere had a radius of 4 mm and its geometrical center was 2 mm away from the origin along the x-axis. The absorption coefficient of the cylinder and the sphere were 0.2 and 0.35 mm^−1^, respectively. The reduced scattering coefficients were 1.05 mm^−1^ and the refractive indices were 1.37 for both media. The light source was placed at the center of the sphere. Using the optimized optical parameter in Table 2, the error was reduced from 0.3951 to 0.2201.

## 4. DISCUSSION AND CONCLUSIONS

We have present a technique for improving the accuracy of the diffusion model by adjusting optical parameters. Instead of using the accurate tissue optical parameters to produce inaccurate result in highly absorbing media, the optimized optical parameters give better accuracy in such media. GA is used for parameter optimization, which effectively avoid the solution to be trapped in a local optima. Our simulation results show significant error reduction for media with different photon absorptions, and the results have little dependency on the light source location. If the actual optical parameters of the medium are known, the Monte Carlo simulation could generate the measurement data as if obtained from the physical phantom or real mouse experiment, so that the optical parameters optimization technique can be performed purely numerically. In the case that the optical properities are unknown, the proposed technique still applies by replacing the Monte Carlo simulation by the actual physical experiment. However, we emphasize that even though the accuracy of the normalized power distribution is improved with this technique, the solution of DA using the optimized optical parameters does not reflect the actual power. Thus, the calibration process is required to convert the normalized power distribution to the actual power. The optical parameter optimization technique permits diffusion model to work under highly absorption media, so that DA works well over a wider range of applications. For the the in vivo optical tomography such as BLT and FMT, the DA photon propagation model for each mouse organ or tissue is improved numerically prior to the reconstruction, the reconstruction algorithm based on the improved DA model is expected to have improved source localization and intensity accuracy.

## Figures and Tables

**Table 1:** GA configration parameters.

Population size	50
Penerations	200
Crossover rate	80%
Mutation rate	10%
Elitism	best 2

**Table 2:** Optical parameters optimization results. The unit was in mm^−1^.

$\mu_{a}$	$\mu_{s}^{\prime }$	$\mu_{a}^{\text{adj}}$	$\mu_{s}^{\prime \text{adj}}$
0.20	1.05	0.375366	0.213276
0.35	1.05	0.423532	0.453873

**Table 3:** Accuracy improvements of DA regarding to different light source locations. d is the distance from the origin, ɛ the error without optical parameter optimization, and ${ɛ}^{\text{adj}}$ the improved error.

d (mm)	$\mu_{a}=0.2$ mm^−1^		$\mu_{a}=0.35$ mm^−1^	
	ɛ	${ɛ}^{\text{adj}}$	ɛ	${ɛ}^{\text{adj}}$
1	0.09	0.0406	0.2361	0.0766
2	0.1665	0.1008	0.3801	0.1393
3	0.3151	0.1066	0.5784	0.14
4	0.3944	0.1074	0.6558	0.1183
5	0.373	0.0947	0.6567	0.1608
